# Association between type 1 diabetes and female sexual dysfunction

**DOI:** 10.1186/s12905-020-00939-1

**Published:** 2020-04-16

**Authors:** Virginia Zamponi, Rossella Mazzilli, Olimpia Bitterman, Soraya Olana, Cristina Iorio, Camilla Festa, Chiara Giuliani, Fernando Mazzilli, Angela Napoli

**Affiliations:** 1 Andrology, Department of Clinical and Molecular Medicine, University of Rome “Sapienza”, Sant’Andrea Hospital, via di Grottarossa 1038, University of Rome ‘Sapienza’, Rome, Italy; 2 Diabetology, Department of Clinical and Molecular Medicine, University of Rome “Sapienza”, Sant’Andrea Hospital, Via di Grottarossa 1038, University of Rome ‘Sapienza’, Rome, Italy

**Keywords:** Female sexual dysfunction, FSFI, Diabetes mellitus, CSII, Diabetes complications, BMI

## Abstract

**Background:**

This study aims to evaluate**:** 1) the prevalence of Female Sexual Dysfunction (FSD) in women affected by type 1 Diabetes Mellitus (DM) and the control group; 2) the correlation between duration of DM, HbA1C levels and sexual life quality; 3) the relationship between different methods of insulin administration and sexual life quality; 4) the correlation between FSD and diabetes complications.

**Methods:**

We selected 33 women with type 1 DM and 39 healthy women as controls. Each participant underwent a detailed medical history and physical examination and completed the 6-item Female Sexual Function Index questionnaire (FSFI-6). In patients affected by type 1 DM, the different methods of insulin administration (Multi Drug Injection - MDI or Continuous Subcutaneous Insulin Infusion - CSII) and the presence of DM complications were also investigated.

**Results:**

The prevalence of FSD (total score ≤ 19) was significantly higher in the type 1 DM group than in the control group (12/33, 36.4% and 2/39, 5.2%, respectively; *p* = 0.010). No statistically significant differences were found regarding FSD according to the presence of complications, method of insulin administration or previous pregnancies.

**Conclusions:**

This study underlined that FSD is higher in women affected by type 1 DM than in healthy controls. This could be due to the diabetic neuropathy/angiopathy and the type of insulin administration. Therefore, it is important to investigate FSD in diabetic women, as well as erectile dysfunction in diabetic men.

## Background

Female Sexual Dysfunction (FSD) is an heterogeneous group of disorders characterized by clinically significant disturbances in sexual response or the experience of sexual pleasure. The physiology of female sexual response includes the integrity of the vascular system and of the sensory and autonomic nervous system. Furthermore, a negative association between dissatisfaction with male partner’s sexual performance and female sexual functioning could exist [1].

Many possible organic factors potentially associated with FSD have been studied so far. Although still limited, currently available data show a correlation between FSD and dysmetabolic conditions such as diabetes mellitus (DM), dyslipidaemia, metabolic syndrome and obesity [2–4].

The impact of DM on the pathophysiology of male sexual dysfunction has been already widely investigated [5]. In the latest guidelines for DM treatment [6], male sexual dysfunction is mentioned among diabetes complications, yet there is no mention of female sexual dysfunction. FSD, due to both complexity and socio-cultural reasons, has been studied with a certain delay compared to male sexual dysfunction.

Diabetes is associated with tissue hypotrophy and sensitivity impairment, as a consequence of the synergetic effects of vasculopathy and neuropathy [7, 8]. Interestingly, a positive association between clitoral vascular resistance (assessed by clitoral ecocolorDoppler ultrasound) and metabolic syndrome (mainly insulin resistance) with decreased sexual arousal, body image concerns, and increased somatised anxiety symptoms was also reported [9].

There are few and discordant data on the prevalence of FSD in diabetic women due to differences in methodology (face-to-face interviews, mail questionnaires, telephone interviews), sample size definition and classification of severity of the disease. The prevalence of type 1 diabetes in Italy is equal to 0.3%; 50.4% of women with type 1 diabetes are of childbearing age (15–45 years) [10].

According to a previous study by Enzlin et al., the prevalence of FSD in women with diabetes is about 30% [11]. Regarding the aetiology, the same study highlights both somatic and psychological components as possible risk factors of FSD, even though psychological factors seem to be prevalent.

Another Italian study, conducted on 595 diabetic women aged between 35 and 70, showed an FSD prevalence of 54%, reaching 64% during menopause [3]. According to an Italian meta-analysis, FSD is more common in diabetic women than in healthy controls; the odds ratio of FSD is 2.27 in type 1 DM and 2.49 in type 2 diabetes, while it is 2.02 when considering diabetes in general [12].

Therefore, DM could determine a significantly negative impact on female sexual function, especially when associated with other risk factors, and could significantly affect quality of life (QoL) and interpersonal relationships [13].

The current work aims to evaluate**:** 1) the prevalence of Female Sexual Dysfunction (FSD) in women affected by type 1 Diabetes Mellitus (DM) and the control group; 2) the correlation between duration of DM, HbA1C levels and sexual life quality; 3) the relationship between different methods of insulin administration and sexual life quality; 4) the correlation between FSD and diabetes complications.

## Methods

### Participants

In the present pilot case-control study, we enrolled 72 women from January 2016 to May 2017. Of these, 33 women with type 1 diabetes attended our outpatient clinic (Endocrinology and Diabetology Unit, Sant’Andrea Hospital) for a regular diabetes check, whilst the other 39 healthy control women were recruited from the administrative and clinical staff of the Hospital. The medical staff informed all participants about the existence of this study; participants voluntarily accepted to participate in the study and all signed informed consent.

All participants met the following inclusion criteria: age 18–45 years; premenopausal status and no menstrual abnormalities; no concomitant pathologies (excluding diabetes complications); no use of other medications (a part of insulin); sexual activity over the last 4 weeks; heterosexual orientation; a stable relationship for at least 1 year; absence of sexual disorders in the male partners; and an interval of at least 1 year from the last pregnancy. All controls reported to be healthy, not to take any pharmacological treatment and without a history of diabetes or gestational diabetes.

All subjects enrolled in the present study underwent a detailed medical history collection and a physical examination including Body Mass Index (BMI Kg/m^2^) calculation; mean age and former pregnancies were considered.

In addition, in patients affected by type 1 DM, the following aspects were considered: a) time of onset and duration of type 1 DM, as well as HbA1C values; b) treatment of DM (i.e. Multi Drug Injection - MDI or Continuous Subcutaneous Insulin Infusion - CSII); and c) the study of DM complications through cardiologic, nephrologic, neurologic and ophthalmologic evaluations.

### Questionnaire

The Italian Female Sexual Function Index-6 (FSFI-6) questionnaire [14, 15] is a validated and reliable short form questionnaire to identify symptoms of sexual dysfunction, with an optimal ability in discriminating FSD, with a 93% sensibility and a 94% specificity. It includes six domains: desire, arousal, lubrication, orgasm, satisfaction and dyspareunia. Each subscale is scored from 0 or 1 (worst possible sexual outcome) to 5 (best possible sexual outcome). The total score ranges from 3 to 30. As suggested by Isidori et al., a total score ≤ 19 was considered to be indicative of sexual dysfunction [16].

The FSFI-6 was self-administered and fulfilled independently by each participant in a dedicated hospital room without any possible influence and/or interference from physicians or other healthcare professionals. A sexological counselling was offered to women with a FSFI-6 total score ≤ 19.

### Data analysis

The mean and standard deviation (SD) was calculated for all measured variables. The Kolmogorov-Smirnov test was used to assess the normality of distribution. Unpaired t tests, Mann-Whitney tests and Fisher exact tests were used to analyse differences in personal and demographic data, as appropriate.

Mann-Whitney tests were used to detect statistical differences between the FSFI-scores of the experimental and the control group. Comparisons of CSII-treated women, MDI-treated women and controls were carried out through Kruskal Wallis tests and post hoc Dunn tests.

Similarly, comparisons between diabetic women without complications, diabetic women with at least one complication and controls were carried out using the Kruskal Wallis and post hoc Dunn tests. The differences in FSD prevalence (referred to as a total FSFI-6 score ≤ 19) were investigated by Fisher’s exact tests.

A Spearman correlation test was carried out between the total scores of the FSFI-6 questionnaire and BMIs of the entire sample. Finally, a logistic regression analysis was performed using FSD as dependent variable, and BMI and diabetes as two different predictors.

A *p value* of 0.05 was considered for the statistical procedures. Statistical analysis was carried out with *GraphPadInStat software* (Version 3.06 for Windows, San Diego, CA, USA).

### Sample-size calculation

On the basis of our previous observations [17], we assumed a female sexual dysfunction rate equal to 51% in diabetic women and 9% in controls; therefore, the recruitment of 34 participants would be required to achieve 80% power, with an estimated α error, 0.05 and β error, 0.2. Statistical analysis was carried out with clincalc.com.

## Results

No significant differences were found among the main basal characteristics regarding patients with type1 DM and control group (Table 1).

**Table 1:** Basal characteristics of Type 1 DM group and Control group

	Type 1 DM (n.33)	Control group (n.39)	***P value***
**Age***(years; mean ± SD, range)*	38.7 ± 5.1 (27–44)	37.6 ± 4.3 (27–44)	0.3355
**Menarche***(years; mean ± SD, range)*	12.4 ± 1.6 (10–16)	12.7 ± 1.2 (11–15)	0.5323
**Marital status***(n.; %)*	20/33 (60.6%)	25/39 (64.1%)	0.8102
**High education level (University, Specialization, Master, PhD)***(n.; %)*	10/33 (30.3%)	11/39 (28.2%)	1.000
**Smoking habits***(n.; %)*	7/33 (21.2%)	13/39 (33.3%)	0.2988
**Alcohol consumers***(n.; %)*	10 /33 (30.3%)	12/39 (30.8%)	1.000
**Physical activity***(n.; %)*	14/33 (42.4%)	19/39 (48.7%)	0.6407
**BMI***(Kg/m2; mean ± SD, range)*	25.1 ± 5.0 (17.9–40.7)	20.9 ± 3.0 (17.6–34.4)	< 0.0001
**Previous pregnancies***(n.; %)*	25/33 (75.8%)	11/39 (28.0%)	0.0001

*DM* Diabetes Mellitus, *BMI* Body Mass Index

### Questionnaires

The results are summarised in Table 2.

**Table 2:** Total score and single items score of FSFI-6 in Type 1 DM group and in Control group

Questionnaires	Type 1 DM (n.33)	Control group (n.39)	***P value***
**Total score**	20.3 ± 5.3	24.8 ± 3.4	< 0.0001
**ITEM - 1**	3.1 ± 0.8	4.1 ± 0.7	< 0.0001
**ITEM - 2**	3.6 ± 1.0	4.1 ± 0.9	0.0156
**ITEM - 3**	3.2 ± 1.6	4.5 ± 0.8	0.0017
**ITEM - 4**	3.4 ± 1.4	4.2 ± 0.9	0.0119
**ITEM - 5**	3.7 ± 1.3	4.2 ± 0.7	0.1119
**ITEM - 6**	3.3 ± 1.0	3.6 ± 1.0	0.2060

*DM* Diabetes Mellitus, *FSFI* Female Sexual Function Index

The prevalence of *FSD* (total score ≤ 19) was significantly higher in the type 1 DM group (12/33, 36.4%; 95% confidence interval [CI] 18–31) compared to the control group (2/39, 5.2%; 95% CI 3–5; *p* = 0.010). The Relative Risk (RR) was 2.4.

Total score was significantly lower in type1 DM compared to the control group (*p* < 0.0001).

Regarding the single items, a significant difference was found for item 1, 2, 3 and 4 (*p* < 0.0001, *p* = 0.0156, *p* = 0.0017 and *p* = 0.0119, respectively).

### BMI

The mean values of BMI in the group of women affected by type 1 DM was significantly higher compared to the control group (25.1 ± 5.0 vs. 20.9 ± 3.0; *p* < 0.0001) (Table 1).

A significant correlation was found between BMI values and FSFI-6 total scores in the whole population (Spearman correlation analysis; *p* = 0.0029).

However, when we divided diabetic patients into three sub-groups according to BMI values kg/m^2^ (BMI < 25 (21.8 ± 2.5), n.19; BMI ≥25 < 30 (27.4 ± 1.5), n.10; BMI ≥ 30, (34.4 ± 4.8), n.4), no significant difference was observed in FSFI-6 mean total scores (FSFI total scores: BMI < 25: 19.9 ± 5.6 vs BMI 25–30: 20.3 ± 4.8 vs BMI > 30: 22.0 ± 6.9, respectively; ANOVA: *p* = n.s).

When a logistic regression analysis was performed using FSD as dependent variable and BMI and diabetes as different predictors, only diabetes remained significant (diabetes, *p* = 0.015; RR 2,4 95% C.I 15.6–3.5; BMI, *P* = 0.32).

### Previous pregnancies

The prevalence of uniparous or multiparous women was higher among women with type 1 DM (25/33; 75.8%) compared to the control group (11/39; 28.0%) (*p* = 0.0001). Nonetheless, a comparison between uniparous/multiparous women and nulliparous women of the type 1 DM group showed no significant differences for any of the FSFI-6 items or total score (Table 3a). Similar results were obtained when comparing uniparous/multiparous women and nulliparous women from the control group (Table 3b).

**Table 3:** Total score and single items of FSFI-6 in uniparous/multiparous women and nulliparous women in: a) Type 1 DM Group; b) Control group

**Questionnaires**	**Uniparous/multiparous Type 1 DM**	**Nulliparous Type 1 DM**	***P value***
Total score	20.9 ± 5.4	18.8 ± 5.2	0.3241
ITEM - 1	3.0 ± 0.8	3.1 ± 1.0	0.5540
ITEM - 2	3.6 ± 1.0	3.4 ± 1.0	0.4999
ITEM - 3	3.4 ± 1.6	2.6 ± 1.7	0.2267
ITEM - 4	3.6 ± 1.4	3.0 ± 1.4	0.2557
ITEM - 5	3.7 ± 1.3	3.6 ± 1.3	0.7841
ITEM - 6	3.3 ± 1.0	3.5 ± 0.5	0.5127
**Questionnaires**	**Uniparous/multiparous Control group**	**Nulliparous Control group**	***P value***
Total score	25.3 ± 2.4	24.7 ± 3.6	0.9708
ITEM - 1	3.9 ± 0.7	4.2 ± 0.7	0.3551
ITEM - 2	4.6 ± 0.5	4.0 ± 1.0	0.1291
ITEM - 3	4.4 ± 0.8	4.5 ± 0.8	0.7234
ITEM - 4	4.4 ± 0.8	4.2 ± 0.9	0.5296
ITEM - 5	4.3 ± 0.5	4.2 ± 0.8	0.9852
ITEM - 6	3.7 ± 0.8	3.6 ± 1.0	0.8827

*DM* Diabetes Mellitus, *FSFI* Female Sexual Function Index

### Onset and duration of DM, HbA1c

The results are summarised in Table 4.

**Table 4:** Onset and duration of DM, HbA1c values and parameters of the metabolic syndrome

	Mean ± SD (range)
**Onset of DM***(Age)*	14.7 ± 8.0 (2–29)
**Duration of DM***(Years)*	24.8 ± 8.0 (4–40)
**HbA1C***(%)*	7.9 ± 1.4 (5.1–11.7)
**HbA1C***(mmol/mol)*	62.7 ± 15 (32–104)
**Total cholesterol***(mg/dl)*	177.4 ± 25.3 (134–229)
**HDL***(mg/dl)*	56.5 ± 16.9 (31–91)
**Triglycerides***(mg/dl)*	60.5 ± 30.4 (30–194)
**LDL***(mg/dl)*	100.5 ± 18.6 (72.2–149.6)
**Blood Pressure Max***(mmHg)*	116.1 ± 15.0 (100–160)
**Blood Pressure Min***(mmHg)*	70.8 ± 6.5 (60–90)

*DM* Diabetes Mellitus

The mean onset age of disease was 14.7 ± 8.0 years. The mean duration of disease was 24.9 ± 8.0 years. In 32/33 subjects (97.0%), the duration of DM was longer than 10 years. No significant correlations were found between the duration of disease and the total score for the FSFI-6 questionnaire.

The mean HbA1C of diabetic patients was 7.9 ± 1.4% (62.7 ± 15 mmol/mol). No statistically significant correlations were found between HbA1c values and total score for the FSFI-6 questionnaire.

### FSD and MDI, CSII

The results are summarized in Table 5a. Among the 33 women with Type 1 DM, 13 (39.4%) were treated with MDI, while 20 subjects (60.6%) with CSII.

**Table 5:** Total score and single items of FSFI-6 in:: a) CSII and MDI-treated women vs Control group; b) in women affected by complicated DM (Complications) or without complications (No complications) vs Control group

**Questionnaires**	**CSII**	**MDI**	**Control group**	***P value***
Total score	21.5 ± 4.2	18.8 ± 6.6	24.8 ± 3.4	0.0004
ITEM - 1	3.2 ± 0.7	2.9 ± 1.0	4.1 ± 0.7	< 0.0001
ITEM - 2	3.9 ± 0.7	3.2 ± 1.1	4.1 ± 0.9	0.0087
ITEM - 3	3.7 ± 1.4	2.6 ± 1.9	4.5 ± 0.8	0.0014
ITEM - 4	3.5 ± 1.4	3.5 ± 1.5	4.2 ± 0.9	0.1255
ITEM - 5	4.1 ± 0.7	3.1 ± 1.7	4.2 ± 0.7	0.1345
ITEM - 6	3.2 ± 1.0	3.6 ± 1.0	3.6 ± 1.0	0.2386
**Questionnaires**	**Complications**	**No complications**	**Control group**	***P value***
Total score	18.1 ± 5.9	22.1 ± 4.1	24.8 ± 3.4	< 0.0001
ITEM - 1	2.9 ± 1.0	3.2 ± 0.6	4.1 ± 0.7	< 0.0001
ITEM - 2	3.3 ± 1.1	3.8 ± 0.8	4.1 ± 0.9	< 0.0133
ITEM - 3	2.5 ± 1.6	3.9 ± 1.4	4.5 ± 0.8	< 0.0002
ITEM - 4	3.1 ± 1.5	3.6 ± 1.3	4.2 ± 0.9	< 0.0257
ITEM - 5	3.1 ± 1.5	4.2 ± 0.8	4.2 ± 0.7	< 0.0296
ITEM - 6	3.2 ± 0.9	3.4 ± 1.0	3.6 ± 1.0	0.2458

*DM* Diabetes Mellitus, *MDI* Multi Daily Infusion, *CSII* Continuous Subcutaneous Insulin Infusion, *FSFI* Female Sexual Function Index

The prevalence of FSD was 30.0% in the CSII group and 38.5% in MDI-treated women (*p* = NS).

The comparison of the MDI-treated group, the CSII-treated group and the control group by Kruskal Wallis test showed a significant difference for the total score, and items 1, 2 and 3 (*p* = 0.0004, *p* < 0.0001, *p* = 0.0087 and *p* = 0.0014, respectively).

Dunn’s post hoc test revealed a significant difference between the MDI group and the control group (*p* < 0.01) and between the CSII group and controls (*p* < 0.05) concerning total score, CSII and controls (*p* < 0.001) and MDI and controls (*p* < 0.001) concerning item1, and MDI and controls for items 2 and 3 (*p* < 0.01).

### Complications of diabetes

Among the 33 women included, 15 (45.5%) showed at least one DM1 complication between neuropathy (*n* = 9/15; 60.0%), retinopathy (*n* = 10/15; 66.7%), nephropathy (*n* = 4/15; 26.7%) and vasculopathy (*n* = 2; 13.3%).

The results are summarised in Table 5b.

FSD prevalence was 46.6% in the group of women affected by complicated diabetes mellitus compared to 27.8% in the group of women with diabetes mellitus without complications (*p* = NS). The comparison of the three groups (complicated DM, not complicated and controls) with the Kruskal Wallis test showed a significant difference for the total score and for items 1, 2, 3, 4 and 5 (*p* < 0.0001, *p* < 0.0001, *p* < 0.0133, *p* < 0.0002, *p* < 0.0257 and *p* < 0.0296, respectively).

Dunn’s post hoc test revealed significant differences between: the group with complications and controls for the total score (*p* < 0.001), item 1 (*p* < 0.001), item 2 (*p* < 0.05), item 3 (*p* < 0.001), item 4 (*p* < 0.05) and item 5 (*p* < 0.05); the group of diabetic women without complications and controls for item 1 (*p* < 0.001) and the groups of women with complicated diabetes and non-complicated diabetes (*p* < 0.05).

## Discussion

The present study aimed to investigate the association between type 1 diabetes mellitus and female sexuality. In recent years, many studies have been carried out regarding FSD [4, 18, 19]; however, as suggested by the meta-analysis of Pontiroli et al. [12], few and inconsistent data about FSD in women affected by Type 1 DM are currently available. Possible explanations for the lack of strong scientific evidence about this topic could be found in the absence of standardisation regarding several studies. Other biases could be represented by small populations, age and BMI variability [20], endocrine disorders [21, 22] and concomitant therapies [23].

According to our previous study [17] and that of Doruk et al. [24], the Type1 diabetic women showed a significantly higher prevalence of FSD compared to the control group. In particular, statistical analysis showed significant differences for total score, item 1 (sexual desire), item 2 (excitation), item 3 (lubrication) and item 4 (frequency of orgasm). No significant differences were found among the sphere of global sexual satisfaction (item 5) and dyspareunia (item 6), even though the mean scores were higher in the control group.

This could be because Type 1 DM is a chronic disease with a negative impact on the QoL [25] which can affect sexual function, representing one of the major components in the QoL of a fertile woman. There are both psychological and organic causes. Regarding psychological aspects, an association between type 1 DM and anxiety-depressive syndromes has been described [11, 26], while organic aspects include hormonal changes [21, 22], a greater risk of genitourinary urinary tract infections [27, 28], the neurotoxic effect of hyperglycaemia and mucous dehydration (resulting in vaginal dryness) associated with DM [29].

Several authors showed a higher risk of FSD in women with a higher BMI [12, 20, 30]. To this regard, some pathogenic hypothesis have been proposed. Firstly, hormonal and inflammatory responses induced by the fatty cell-secreted cytokine factors (i.e. TNF-alpha, IL-6and leptin) may contribute to the onset of sexual dysfunction [31]. Furthermore, an increased BMI could determine physical impairment and psychological disorders affecting the quality of sexual life, which is strongly related to the perception of body image [32].

In our study, we highlighted a significant correlation between BMI values and total score of the FSFI-6 questionnaire considering overall diabetic and healthy women. However, when we subdivided diabetic patients according to BMI values, we did not find any significant difference in FSFI-6 total scores (mean ± SD) between the three groups; this finding could depend on the small number of obese women with a moderate/severe obesity of our study group. Furthermore, a logistic regression analysis performed using FSD as dependent variable and BMI and diabetes as different predictors, only diabetes remained significant. Considering these results, type1 DM seems to be the main risk factor for FSD in young fertile women.

Regarding the type of insulin administration, we observed a higher prevalence of FSD in women with MDI administration compared to CSII, both for total score and single items, showing better sexual outcomes in the second group, even though the results are not statistically significant. These results are in agreement to those of Maiorino et al. [4], showing that women with type 1 DM and CSII therapy have a lower prevalence of FSD than women undergoing MDI therapy. This could be due a reduced variability of the glycaemic profile in patients with CSII compared to MDI, as suggested by Reddy et al. [33].

The CSII group showed a prevalence of FSD similar to that of the healthy population.

Regarding the possible relationship with diabetes complications, according to previous studies [26], we observed a higher prevalence of FSD in women with complicated diabetes compared to uncomplicated diabetes. As pathogenic factors, diabetic neuropathy (mostly the sensory component) could affect genital sexual response to tactile stimulation [34], while diabetic angiopathy could lead to hypotrophy of clitoral erectile structures (resulting in a reduced response to sexual stimulation) and a reduction or lack of vaginal lubrication (with greater risk of dyspareunia) [7].

To this regard, recent studies highlighted that the peripheral sexual response in female is a vascular-dependent event and the vascular function of the genital tract could be affected by common cardiometabolic alterations [35, 36].

It is not surprising that item 3, vaginal lubrication, was the one which showed the greatest differences within the study sample: not only between diabetic women and controls, but also between women with complicated and uncomplicated DM. Other complications, such as nephropathy [37], retinopathy, foot ulcers [38, 39] and autonomic heart disease [40, 41], may also have a negative effect on the overall quality of life and, indirectly, on sexual activity.

One of the most important studies about this aspect was by Enzlin et al. [26], which referred to a large cohort study on FSD in type 1 diabetic women. The author highlighted a significant association between FSD and diabetic microangiopathy.

Despite there is evidence that nulliparous women show superior sexual function scores compared with parous women [42], we did not find any association between parity and FSFI-6 scores inside each single study group. This could be due to the small number and to the differences in the prevalence of uniparous/multiparous women between the study group and the control group. Moreover, we explored FSFI in young women after more than 1 year from their last pregnancy.

Finally, the assessment of female sexual function only by using a questionnaire and the lack of hormonal parameters and clitoral ecolorDoppler ultrasound of the study population represented the limits of this study. Future and larger studies on hormonal, clinical and instrumental aspects as well as on psycho-relational components will help in understanding the role of these factors on sexual function of fertile women with type1 diabetes.

## Conclusions

In conclusion, this study underlined that FSD is higher in women affected by type 1 DM than in healthy controls. This could be due to the diabetic neuropathy/angiopathy and the type of insulin administration. Therefore, it should be very important to investigate FSD in diabetic women, as well as erectile dysfunction in diabetic males.

## Data Availability

The datasets used and/or analysed during the current study are available from the corresponding author on reasonable request.

## Abbreviations

FSD: Female Sexual Dysfunction
DM: Diabetes Mellitus
Qol: Quality Of Life
BMI: Body Mass Index
MDI: Multi Drug Injection
CSII: Continuous Subcutaneous Insulin Infusion
SD: Standard Deviation
FSFI: Female Sexual Function Index
